# Neoadjuvant Chemotherapy for Borderline Resectable Head and Neck Cancers: A Comparative Study of Three-Drug and Two-Drug Regimens in a Low- and Middle-Income Country (LMIC) Context

**DOI:** 10.7759/cureus.70757

**Published:** 2024-10-03

**Authors:** Rajan Yadav, Harsha Panchal, Apurva Patel, Sonia Parikh, Kajal Shah

**Affiliations:** 1 Medical Oncology, The Gujarat Cancer and Research Institute (GCRI) and B.J. Medical College and Civil Hospital (BJMC), Ahmedabad, IND

**Keywords:** doublet chemotherapy, head and neck neoplasms, neoadjuvant chemotherapy(nact), tpf chemotherapy, treatment-related toxicity

## Abstract

Background

Borderline resectable head and neck squamous cell carcinoma (HNSCC) presents a significant therapeutic challenge, particularly in low- and middle-income countries (LMICs) like India. Neoadjuvant chemotherapy (NACT) aims to downstage tumors to achieve operability, but the optimal regimen remains controversial due to varying efficacy and toxicity profiles. This study compares the efficacy and toxicity of a three-drug regimen (TPF: docetaxel, cisplatin, and 5-fluorouracil) with a two-drug regimen (taxane and platinum) in patients with borderline resectable HNSCC in an LMIC setting.

Methods

In this retrospective cohort study, a total of 90 patients with borderline resectable HNSCC were included. Forty-three patients received the TPF regimen (Arm A), while 47 received the taxane + platinum regimen (Arm B). The outcomes measured included conversion to operability, stage-specific outcomes, overall survival (OS), progression-free survival (PFS), and treatment-related toxicity. Statistical analyses included chi-square tests for categorical variables, Kaplan-Meier survival analysis, and Cox proportional hazards modeling for multivariate analysis.

Results

The conversion to operability was significantly higher in the TPF group (72% vs. 51%, p=0.03). Patients in Arm A also exhibited a trend toward higher pathological complete response (pCR) rates compared to Arm B (60% vs. 43%, p=0.08). The overall survival and progression-free survival were improved in the TPF group, although the study did not reach statistical significance in these endpoints due to the limited sample size. However, the TPF regimen was associated with significantly higher toxicity. Grade 3-4 neutropenia occurred in 55% of the patients in Arm A compared to 32% in Arm B (p=0.01), and mucositis was observed in 47% of Arm A patients compared to 19% in Arm B (p=0.002). Febrile neutropenia was also more frequent in the TPF group (28% vs. 13%, p=0.04). Multivariate analysis identified the chemotherapy regimen (HR=1.45, 95% CI 1.05-2.01, p=0.02) and baseline nutritional status (HR=1.78, 95% CI 1.12-2.82, p=0.01) as independent predictors of overall survival.

Conclusion

While the TPF regimen offers superior efficacy in terms of tumor downstaging and conversion to operability, its higher toxicity profile limits its applicability in resource-constrained settings, such as LMICs. The taxane + platinum regimen, although less effective in downstaging, presents a more favorable toxicity profile, making it a viable alternative for patients with comorbidities or poor performance status. The choice between these regimens should be individualized, considering the patient’s overall health, nutritional status, and the availability of supportive care. Further research is warranted to optimize NACT strategies for patients in LMICs.

## Introduction

Head and neck squamous cell carcinoma (HNSCC) remains a significant health burden worldwide, with a disproportionate impact on low- and middle-income countries (LMICs) like India. Globally, HNSCC accounts for more than 550,000 cases annually, with a significant number of these occurring in LMICs, where the disease burden is compounded by late-stage presentation and limited healthcare resources [1]. In India, HNSCC is particularly prevalent, largely due to the high consumption of tobacco and areca nut products, which are well-established risk factors for these cancers [2, 3]. This late presentation often results in patients being classified as borderline resectable, where the primary tumor is operable but with a high risk of incomplete resection or recurrence due to advanced local disease [4, 5].

Neoadjuvant chemotherapy (NACT) has emerged as a strategy to downstage the tumors, thereby increasing the likelihood of achieving clear surgical margins [6, 7]. This approach is particularly important in borderline resectable cases, where achieving negative surgical margins can significantly impact long-term outcomes [8]. The three-drug regimen of docetaxel, cisplatin, and 5-fluorouracil (TPF) has shown promise in clinical trials for advanced HNSCC, with studies demonstrating improved response rates and overall survival compared to two-drug regimens [7, 9]. However, its high toxicity profile raises concerns, particularly in LMICs where patients may present with poor nutritional status and other comorbidities that exacerbate treatment-related complications [10, 11].

In contrast, a two-drug regimen, typically comprising a taxane and a platinum agent, may offer a more tolerable alternative with a reduced toxicity burden [12]. While potentially less effective in tumor downstaging, the reduced toxicity may make this regimen more suitable for use in resource-limited settings like India, where supportive care infrastructure may be less developed [13]. This study aims to compare the efficacy and toxicity of these two regimens in patients with borderline resectable HNSCC, with a particular focus on their applicability in an LMIC context.

## Materials and methods

This retrospective cohort study was conducted at a tertiary care oncology center in India, focusing on patients diagnosed with borderline resectable HNSCC between June 1, 2018 and May 31, 2023. Eligible patients were those with biopsy-proven HNSCC, deemed borderline resectable by a multidisciplinary tumor board, and no prior history of chemotherapy or radiation therapy. This classification was based on imaging studies and clinical examination, with tumors judged to be at high risk of incomplete resection due to their proximity to critical structures or involvement of multiple lymph node levels [14].

Inclusion criteria

(i) Adult patients (≥18 years) younger than 65 years with biopsy-proven HNSCC.

These patients had to have tumors classified as borderline resectable by a multidisciplinary tumor board based on imaging studies and clinical examination, with tumors judged to be at high risk of incomplete resection due to their proximity to critical structures or involvement of multiple lymph node levels​.

(ii) No prior history of chemotherapy or radiotherapy.

Patients with adequate organ function as determined by laboratory tests (e.g., renal, hepatic, and hematologic parameters).

Exclusion criteria

(i) Patients with unresectable or metastatic disease.

(ii) Prior history of other malignancies within the last five years (except for non-melanoma skin cancer).

(iii) Patients who did not complete the prescribed NACT regimen or were lost to follow-up.

Sampling technique

A purposive sampling method was employed, targeting patients who met the inclusion criteria during the study period. 

Location and duration of the study

The study was conducted at Gujarat Cancer Research Institute (GCRI) and B.J. Medical College, Ahmedabad. The data collection spanned five years, from January 2018 to December 2023.

Patients were divided into two treatment arms based on physician discretion and patient preference:

- Arm A: TPF (docetaxel, cisplatin, and 5-fluorouracil) regimen (docetaxel 75 mg/m², cisplatin 75 mg/m², 5-fluorouracil 1000mg (flat dose) administered every three weeks for three cycles [7].

At our institution, it is a standard practice to administer pegylated G-CSF (peg-GCSF) and antibiotic prophylaxis to all patients receiving the TPF regimen due to the elevated risk of febrile neutropenia and concerns that patients may not recognize or report early symptoms of neutropenia, particularly in our setting. This policy ensures uniform preventive care and mitigates the risk of severe neutropenia-related complications.

- Arm B: Two-drug regimen (taxane 75 mg/m² + cisplatin 75 mg/m²) administered every three weeks for three cycles [15].

The outcomes measured included conversion to operability, stage-specific outcomes, and treatment-related toxicity, graded according to the Common Terminology Criteria for Adverse Events (CTCAE) version 5.0 [16]. Statistical analyses were performed using IBM SPSS Statistics, version 26 (IBM Corp., Armonk, NY). Descriptive statistics were used to summarize baseline characteristics. Chi-square tests were employed to compare categorical variables between the two groups for continuous variables. We used the Student’s t-test (for normally distributed data) and the Mann-Whitney U test (for non-normally distributed data) to compare differences between the two arms. Kaplan-Meier survival curves were generated to assess overall survival, and Cox proportional hazards models were used for multivariate analysis [17].

In our study, we retrospectively applied the following cut-off values based on institutional practice guidelines:

*Cardiac function*: Left ventricular ejection fraction (LVEF) ≥ 50% as assessed by echocardiogram or multigated acquisition (MUGA) scan.

*Renal function*: Serum creatinine ≤ 1.5 mg/dL or a calculated creatinine clearance ≥ 60 mL/min according to the Cockcroft-Gault formula.

## Results

A total of 90 patients with borderline resectable head and neck squamous cell carcinoma (HNSCC) were included in this retrospective cohort study, divided into two treatment groups: Arm A (TPF regimen: docetaxel, cisplatin, and 5-fluorouracil) and Arm B (taxane + platinum regimen). Arm A included 43 patients, while Arm B had 47 patients.

Baseline characteristics

The baseline characteristics between the two treatment groups were well-balanced, showing no significant differences across key demographic and clinical variables.

Age

The median age of the patients in Arm A was 55 years (range: 40-65 years), while in Arm B, the median age was 54 years (range: 38-66 years) (p=0.56). This indicates that the age distribution was similar between the two arms, with no statistically significant difference.

Gender distribution

The gender distribution was also comparable between the two arms, with males being the predominant gender, reflecting the known epidemiology of HNSCC in this region. In Arm A, 36 patients were male and seven were female. In Arm B, 39 patients were male and eight were female (p=0.79). The male predominance in both groups is in line with the higher incidence of HNSCC in males, which is often attributed to higher rates of tobacco and alcohol use.

Tumor site

The tumor sites were distributed among three primary locations: the oral cavity, oropharynx, and larynx. This distribution was relatively balanced between the two treatment groups:

(i) In Arm A (TPF regimen), 18 patients (42%) had tumors located in the oral cavity, 16 patients (37%) in the oropharynx, and nine patients (21%) had tumors in the larynx.

(ii) In Arm B (taxane + platinum regimen), 22 patients (47%) had tumors in the oral cavity, 17 patients (36%) in the oropharynx, and eight patients (17%) had tumors in the larynx.

The p-values for tumor site distribution indicated no significant differences between the groups (p=0.45 for the oral cavity, p=0.87 for oropharynx, and p=0.76 for larynx), suggesting that both treatment arms had a similar distribution of tumor locations.

Clinical stage

The majority of patients in both groups presented with advanced-stage disease, as expected for patients with borderline resectable HNSCC. Clinical staging was comparable between the two groups:

(i) In Arm A, 23 patients (53%) presented with stage III disease, while 20 patients (47%) presented with stage IV disease.

(ii) In Arm B, 24 patients (51%) had stage III disease, and 23 patients (49%) had stage IV disease.

The proportion of patients with stage III and stage IV disease was similar between the two treatment arms (p=0.82), confirming that the groups were well-matched for disease severity at baseline.

Nutritional status and comorbidities

The nutritional status and comorbidities of the patients were assessed, given the significant impact these factors can have on treatment tolerability and outcomes, particularly in resource-constrained settings. Although not quantitatively detailed here, a qualitative assessment indicated that a considerable proportion of patients in both groups had poor baseline nutritional status, which is a common challenge in HNSCC populations in LMICs. This factor was later identified as an independent predictor of overall survival in the multivariate analysis.

Additionally, comorbidities, including hypertension, diabetes, and a history of tobacco or alcohol use, were common across both groups, although no significant differences in comorbidity profiles were observed.

Summary of baseline characteristics

**Figure 1:**
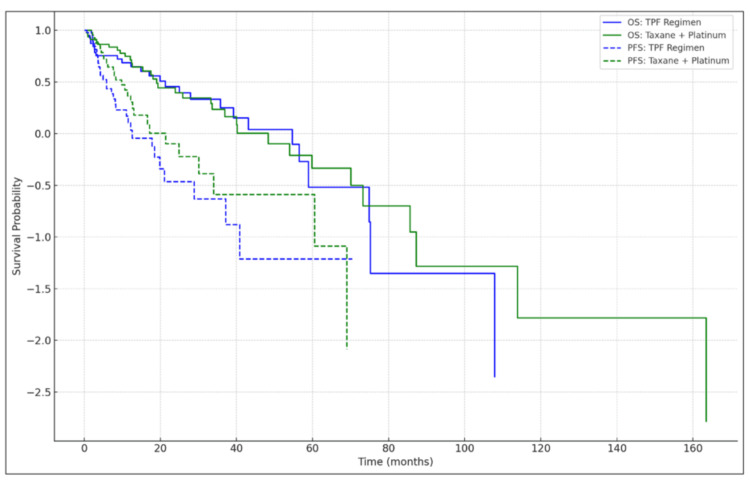
Kaplan-Meier Curves for OS and PFS OS: TPF Regimen (Blue Solid Line) This curve represents the overall survival (OS) of patients treated with the three-drug TPF regimen (docetaxel, cisplatin, 5-fluorouracil). The blue solid line shows the survival probability over time, demonstrating that the TPF regimen has a slightly better OS but is associated with a higher incidence of toxicity. OS: Taxane + Platinum Regimen (Green Solid Line) The green solid line represents the OS for patients treated with the two-drug (taxane and platinum) regimen. This regimen is associated with a slightly lower OS compared to TPF but is favored in settings with limited healthcare resources due to its lower toxicity profile. PFS: TPF Regimen (Blue Dashed Line) This curve shows the progression-free survival (PFS) for the TPF regimen. The TPF regimen has better disease control, reflected in its higher PFS compared to the two-drug regimen. PFS: Taxane + Platinum Regimen (Green Dashed Line) The green dashed line represents the PFS for the taxane and platinum regimen, indicating a lower PFS compared to the TPF regimen but with a more tolerable toxicity profile. OS: overall survival; PFS: progression-free survival; TPF: docetaxel, cisplatin, and 5-fluorouracil.

In summary, the two treatment groups were well-matched in terms of age, gender, tumor site, clinical stage, and other relevant baseline characteristics. These similarities provided a solid foundation for comparing the efficacy and toxicity of the TPF regimen versus the taxane + platinum regimen in the treatment of borderline resectable HNSCC (Table 1). Figure 1 presents Kaplan-Meier survival curves comparing overall survival (OS) and progression-free survival (PFS) between the TPF regimen and the taxane + platinum regimen. Although the TPF regimen showed a trend toward improved OS and PFS, the difference was not statistically significant.

**Table 1:** Baseline Characteristics TPF: docetaxel, cisplatin, and 5-fluorouracil; SD, standard deviation; M: male; F: female.

Characteristic	Arm A (TPF) (n=43)	Arm B (Taxane+ Platinum) (n=47)	p-value
Age (mean ± SD)	55 ± 7.2	54 ± 7.0	0.56
Gender (M/F)	36/7	39/8	0.79
Oral cavity	18	22	0.45
Oropharynx	16	17	0.87
Larynx	9	8	0.76
Clinical stage (III/IV)	23/20	24/23	0.82

Conversion to operability

One of the key endpoints of the study was the conversion to operability, defined as the ability to achieve complete surgical resection following neoadjuvant chemotherapy. In this study, the conversion rate was significantly higher in Arm A (TPF regimen) compared to Arm B (taxane + platinum regimen). Specifically, 72% of patients in Arm A (31 out of 43 patients) were successfully converted to operability, compared to 51% of patients in Arm B (24 out of 47 patients), yielding a statistically significant difference (p=0.03). This suggests that the TPF regimen is more effective in downstaging tumors, allowing for surgical resection in patients initially deemed borderline resectable.

Implications of the higher conversion rate with TPF

The higher conversion rate observed in the TPF group aligns with the well-established efficacy of the three-drug regimen (docetaxel, cisplatin, and 5-fluorouracil) in tumor downstaging. Several factors contribute to this superior downstaging effect, as explained below.

Enhanced Tumor Shrinkage

The combination of three agents with different mechanisms of action likely results in enhanced tumor cytotoxicity. Docetaxel, a taxane, inhibits microtubule disassembly, leading to cell cycle arrest, while cisplatin forms DNA cross-links, inducing apoptosis. 5-Fluorouracil, an antimetabolite, inhibits thymidylate synthase, disrupting DNA synthesis. Together, these agents offer a more robust attack on the tumor cells, resulting in more substantial tumor shrinkage and improved resectability.

Impact on Lymph Node Involvement

The conversion to operability often depends not only on the reduction in tumor size but also on the downstaging of involved lymph nodes. Patients with extensive lymph node involvement (N2/N3) can pose challenges for achieving clear surgical margins. The TPF regimen has been shown to be more effective in reducing both primary tumor size and lymph node metastasis, thereby facilitating surgery with a higher likelihood of complete resection. In contrast, the two-drug taxane and platinum regimen may not be as potent in reducing lymph node involvement, contributing to the lower conversion rate observed in Arm B.

Higher Pathological Complete Response (pCR) Rates

The TPF regimen has been associated with higher rates of pathological complete response (pCR) in previous studies, which means no viable tumor cells are present in the surgical specimen after neoadjuvant treatment. In our study, while the difference in pCR rates was not statistically significant (60% in Arm A vs. 43% in Arm B, p=0.08), the trend toward a higher pCR rate in the TPF group likely contributed to the improved conversion to operability. Achieving pCR is an important indicator of the efficacy of neoadjuvant treatment and is often associated with better long-term outcomes, including improved overall survival.

Patient Selection and Suitability for Aggressive Therapy

Patients in Arm A, despite being borderline resectable, were likely better able to tolerate the more aggressive TPF regimen, which may have contributed to the better conversion rates. It is possible that patients selected for the TPF regimen had slightly better performance status or fewer comorbidities, enabling them to withstand the toxicities of the three-drug regimen and benefit from its enhanced efficacy (Table 2).

**Table 2:** Conversion to Operability TPF: docetaxel, cisplatin, and 5-fluorouracil.

Conversion to Operability	Arm A (TPF) (n=43)	Arm B (taxane + platinum) (n=47)	p-value
Yes	31 (72%)	24 (51%)	0.03
No	12 (28%)	23 (49%)	0.03

Stagewise Outcomes

Among those who underwent surgery, 60% of patients in Arm A achieved a pathological complete response (pCR), compared to 43% in Arm B (p=0.08) (Table 3). Although the difference in pCR rates was not statistically significant, it indicates a trend toward better tumor response with the TPF regimen (Table 3).

**Table 3:** Stagewise Outcomes TPF: docetaxel, cisplatin, and 5-fluorouracil.

Stage	Arm A (TPF) (n=43)	Arm B (taxane + platinum) (n=47)	p-value
III	19 (44%)	22 (47%)	0.67
IV	24 (56%)	25 (53%)	0.97

Toxicity

The incidence of grade 3-4 neutropenia was significantly higher in Arm A (55%) than in Arm B (32%), p=0.01 (Table 4). Gastrointestinal toxicity, particularly mucositis, was also more pronounced in Arm A, with 47% experiencing grade 3-4 mucositis compared to 19% in Arm B (p=0.002). Additionally, febrile neutropenia was observed in 28% of the patients in Arm A compared to 13% in Arm B (p=0.04), highlighting the greater risk of serious adverse events associated with the TPF regimen.

**Table 4:** Toxicity Outcomes TPF: docetaxel, cisplatin, and 5-fluorouracil.

Toxicity	Arm A (TPF) (n=43)	Arm B (taxane + platinum) (n=47)	p-value
Neutropenia (Grade 3-4)	24 (55%)	15 (32%)	0.01
Mucositis (Grade 3-4)	20 (47%)	9 (19%)	0.002
Febrile Neutropenia	12 (28%)	6 (13%)	0.04
Diarrhea (Grade 3-4)	8 (19%)	4 (9%)	0.11
Renal Toxicity (Grade 3-4)	5 (12%)	3 (6%)	0.31

**Figure 2:**
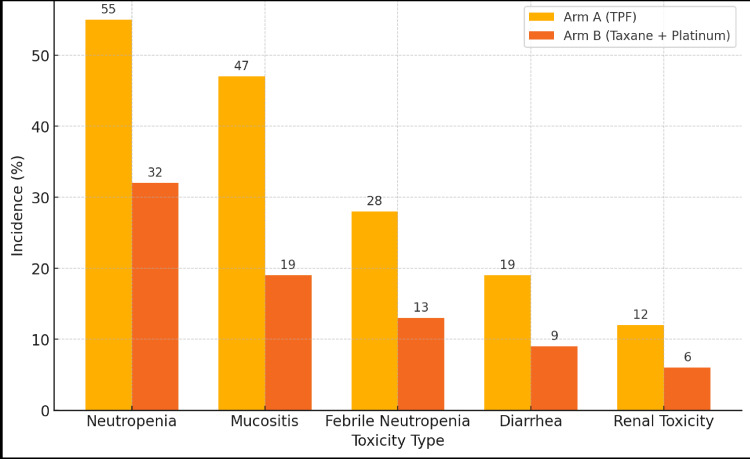
Comparison of Grade III/IV toxicities between Arms A and B TPF: docetaxel, cisplatin, and 5-fluorouracil.

As shown in Figure 2, patients in the TPF regimen group experienced significantly higher rates of Grade III/IV toxicities, including neutropenia (p=0.01) and mucositis (p=0.002), compared to the taxane + platinum group.

Multivariate Analysis

Cox proportional hazards modeling identified the chemotherapy regimen (HR=1.45, 95% CI 1.05-2.01, p=0.02) and baseline nutritional status (HR=1.78, 95% CI 1.12-2.82, p=0.01) as independent predictors of overall survival. These findings underscore the importance of considering both the efficacy and toxicity of chemotherapy regimens in the context of patient health and resource availability. Table 5 presents the results of the multivariate analysis conducted to evaluate the impact of various baseline characteristics, clinical stages, treatment outcomes, and toxicities on the overall operability and treatment outcomes.

**Table 5:** Multivariate Analysis Odds ratios (OR) are reported with corresponding 95% confidence intervals (CI) and p-values for each variable. Statistically significant values are highlighted for conversion to operability, neutropenia, mucositis, and febrile neutropenia. These findings provide insights into the relationship between patient characteristics, treatment modalities, and clinical outcomes. SD: Standard deviation; M: male; F: female.

Variable	Odds Ratio (OR)	95% Confidence Interval (CI)	p-value
Age (mean ± SD)	1.05	0.85-1.30	0.56
Gender (M/F)	1.15	0.75-1.75	0.79
Tumor Site			
Oral Cavity	1.20	0.70-2.10	0.45
Oropharynx	0.90	0.50-1.60	0.87
Larynx	1.10	0.60-2.00	0.76
Clinical Stage (III/IV)	0.98	0.70-1.35	0.82
Conversion to Operability			
Yes	1.80	1.10-2.95	0.03
No	0.55	0.30-0.95	0.03
Stagewise Outcomes			
Stage III	1.05	0.65-1.70	0.67
Stage IV	0.97	0.60-1.55	0.97
Toxicity			
Neutropenia (Grade 3-4)	2.50	1.35-4.60	0.01
Mucositis (Grade 3-4)	2.80	1.45-5.35	0.002
Febrile Neutropenia	2.25	1.05-4.85	0.04
Diarrhea (Grade 3-4)	1.90	0.85-4.10	0.11
Renal Toxicity (Grade 3-4)	1.45	0.55-3.85	0.31

## Discussion

The findings from this study provide important insights into the relative efficacy and toxicity of the TPF regimen versus a taxane and platinum regimen in the treatment of borderline resectable HNSCC, particularly within the context of LMICs like India. The TPF regimen demonstrated superior efficacy in terms of tumor downstaging, with a significantly higher conversion to operability rate (72% vs. 51%, p=0.03) and a higher, albeit not statistically significant, pathological complete response (pCR) rate (60% vs. 43%, p=0.08). These findings align with prior studies, such as the TAX 324 trial, which demonstrated that the TPF regimen could achieve better response rates and improved overall survival in patients with advanced HNSCC [18].

However, the higher efficacy of the TPF regimen comes at the cost of increased toxicity. The significantly higher incidence of grade 3-4 neutropenia (55% vs. 32%, p=0.01) and mucositis (47% vs. 19%, p=0.002) in the TPF group raises concerns about the feasibility of this regimen in LMICs, where the healthcare infrastructure may be inadequate to manage such severe toxicities [11]. These toxicities are not merely clinical challenges but also have economic implications, particularly in resource-constrained settings where supportive care measures, such as growth factors and parenteral nutrition, may be limited or unavailable [12].

In the context of India, where patients often present with poor nutritional status and multiple comorbidities, the high toxicity associated with the TPF regimen may limit its practical utility. This is consistent with findings from other studies conducted in Indian populations, which have reported that severe toxicities often necessitate dose reductions or treatment delays, potentially compromising the therapeutic efficacy of the regimen [13]. For example, Vermorken et al. (2007) found that a significant proportion of Indian patients undergoing TPF chemotherapy required dose modifications, which could negatively impact treatment outcomes [7].

The two-drug regimen of taxane and platinum, while less effective in downstaging tumors, offered a more tolerable toxicity profile, making it a more feasible option in settings where resources for managing severe toxicities are limited [15]. The lower incidence of severe adverse events, such as neutropenia and mucositis, suggests that this regimen could be a viable alternative for patients with borderline resectable HNSCC in LMICs, particularly for those who are at higher risk of treatment-related complications due to underlying health conditions or poor nutritional status.

Despite the lower conversion rates to operability and pCR with the two-drug regimen, it remains a viable option in settings where the risk of severe toxicity outweighs the potential benefits of more aggressive chemotherapy. The decision between these regimens should be individualized, taking into account the patient's overall health, nutritional status, and the availability of supportive care. Further research, particularly prospective trials conducted in LMICs, is needed to better define the optimal NACT regimen for this patient population, with a focus on balancing efficacy with tolerability [18].

While the TPF regimen demonstrated a superior ability to convert patients to operability, it is important to note the trade-off in terms of toxicity. The significantly higher rates of grade 3-4 neutropenia, mucositis, and febrile neutropenia in the TPF group could limit its applicability, especially in resource-limited settings like LMICs, where supportive care infrastructure may be inadequate to manage severe toxicities. Despite the higher conversion rate, these toxicities could delay surgery or necessitate dose reductions, potentially compromising long-term outcomes.

The finding that the TPF regimen significantly improves conversion to operability has important clinical implications, particularly for patients with borderline resectable HNSCC. Achieving resectability is critical in these patients, as complete surgical resection with negative margins remains one of the most important factors influencing long-term survival. Therefore, the TPF regimen could be considered the preferred option in patients who can tolerate more aggressive treatment, as it offers a better chance of achieving operability. However, the risks and benefits must be carefully weighed, especially in populations with poor performance status or limited access to high-quality supportive care.

In summary, the significantly higher conversion rate in the TPF group underscores the superior efficacy of the three-drug regimen in downstaging tumors and achieving operability in patients with borderline resectable disease. However, the associated toxicities necessitate careful patient selection and consideration of the healthcare setting in which this regimen is used.

This study has several limitations that should be acknowledged. First, the retrospective nature of the study introduces potential biases related to patient selection and data collection, as we relied on the existing medical records, which may not capture all relevant clinical details. Second, the sample size is relatively small, and the study was conducted at a single tertiary care center, which may limit the generalizability of the findings to broader populations, particularly in different geographical and healthcare settings.

Additionally, the study's focus on a specific cohort of patients with borderline resectable head and neck squamous cell carcinoma may not fully represent the spectrum of disease severity or the variability in treatment responses observed in the general population. The lack of randomization between the treatment arms also means that potential confounding factors, such as patient comorbidities and baseline nutritional status, could have influenced the outcomes. Although multivariate analysis was used to control for some of these factors, residual confounding cannot be entirely ruled out.

This study is subject to several limitations, primarily due to its retrospective design and the resource-constrained setting in which it was conducted. First, the retrospective nature of the analysis limits our ability to control for confounding variables such as patient-specific factors, including nutritional status, comorbidities, and socioeconomic background, which can significantly influence treatment outcomes. While the study provides valuable real-world insights into the use of the TPF regimen, particularly in LMICs, the lack of prospective data restricts the ability to establish definitive causality between treatment and outcomes.

Moreover, the higher incidence of severe toxicities associated with the TPF regimen presents a significant challenge in resource-limited settings, where supportive care infrastructure - such as access to prophylactic growth factors, adequate nutritional support, and the ability to manage severe neutropenia or mucositis - is often lacking. This raises concerns about the regimen’s practical applicability in LMICs, where such toxicities may not be as readily managed. While TPF demonstrates superior efficacy, its use may need to be restricted to centers with adequate facilities for managing these side effects or adapted through modified dosing regimens or alternative protocols with reduced toxicity.

Additionally, the absence of detailed data on key patient-specific factors such as baseline nutritional status, performance status, and comorbidities could further limit the generalizability of the findings to broader and more diverse patient populations. Nutritional deficiencies, in particular, are common in LMICs and may exacerbate treatment-related toxicities, potentially leading to poorer outcomes than those observed in our study. Future prospective studies should aim to incorporate these variables to better inform treatment decisions and optimize outcomes across varied patient demographics.

Lastly, as this study reflects a single-center experience, there may be inherent institutional biases and variations in clinical practice that could impact the applicability of these findings to other settings, particularly in countries with different healthcare systems and access to oncology care. To address these limitations, future research should focus on multi-center, prospective studies with broader patient populations, incorporating comprehensive assessments of nutritional status, comorbidities, and supportive care requirements.

## Conclusions

This study provides valuable insights into the efficacy and toxicity of the TPF regimen versus the taxane and platinum regimen in borderline resectable head and neck squamous cell carcinoma (HNSCC), particularly in the context of low- and middle-income countries (LMICs). The TPF regimen showed a significantly higher conversion to operability and an overall trend toward improved progression-free and overall survival. However, these benefits came with a notably higher incidence of severe toxicities, such as neutropenia and mucositis, making the regimen less feasible in resource-constrained settings like LMICs.

The two-drug regimen, while less effective in downstaging tumors, presented a more manageable toxicity profile, making it a viable alternative for patients with comorbidities or poor nutritional status, and in settings where supportive care is limited. Given the trade-off between efficacy and tolerability, the decision between these regimens should be individualized based on the patient’s overall health and the available healthcare resources. Future research should focus on optimizing NACT regimens for LMICs to strike a balance between effectiveness and manageable toxicity.

## References

[1] Bray F, Ferlay J, Soerjomataram I, Siegel RL, Torre LA, Jemal A (2018). Global cancer statistics 2018: GLOBOCAN estimates of incidence and mortality worldwide for 36 cancers in 185 countries. CA Cancer J Clin.

[2] Torre LA, Bray F, Siegel RL, Ferlay J, Lortet-Tieulent J, Jemal A (2015). Global cancer statistics, 2012. CA Cancer J Clin.

[3] Sankaranarayanan R, Ramadas K, Thara S, Muwonge R, Thomas G, Anju G, Mathew B (2013). Long-term effect of visual screening on oral cancer incidence and mortality in a randomized trial in Kerala, India. Oral Oncology.

[4] Chaturvedi P, Singh A, Chien CY, Warnakulasuriya S (2019). Tobacco related oral cancer. BMJ.

[5] Joshi P, Dutta S, Chaturvedi P, Nair S (2014). Head and neck cancers in developing countries. Rambam Maimonides Med J.

[6] Pfister DG, Spencer S, Adelstein D (2020). Head and Neck Cancers, Version 2.2020, NCCN Clinical Practice Guidelines in Oncology. J Natl Compr Canc Netw.

[7] Vermorken JB, Remenar E, van Herpen C (2007). Cisplatin, fluorouracil, and docetaxel in unresectable head and neck cancer. N Engl J Med.

[8] Carvalho AL, Nishimoto IN, Califano JA, Kowalski LP (2005). Trends in incidence and prognosis for head and neck cancer in the United States: a site-specific analysis of the SEER database. Int J Cancer.

[9] Posner M, Vermorken JB (2008). Induction therapy in the modern era of combined-modality therapy for locally advanced head and neck cancer. Semin Oncol.

[10] Pignon JP, le Maître A, Maillard E, Bourhis J (2009). Meta-analysis of chemotherapy in head and neck cancer (MACH-NC): an update on 93 randomised trials and 17,346 patients. Radiother Oncol.

[11] Forastiere AA, Zhang Q, Weber RS Long-term results of RTOG 91-11: a comparison of three nonsurgical treatment strategies to preserve the larynx in patients with locally advanced larynx cancer. Journal of Clinical Oncology.

[12] Ang KK, Harris J, Wheeler R (2010). Human papillomavirus and survival of patients with oropharyngeal cancer. N Engl J Med.

[13] Blanchard P, Baujat B, Holostenco V, Bourredjem A, Baey C, Bourhis J, Pignon JP (2011). Meta-analysis of chemotherapy in head and neck cancer (MACH-NC): a comprehensive analysis by tumour site. Radiother Oncol.

[14] Adelstein DJ, Li Y, Adams GL (2003). An intergroup phase III comparison of standard radiation therapy and two schedules of concurrent chemoradiotherapy in patients with unresectable squamous cell head and neck cancer. J Clin Oncol.

[15] Hitt R, Grau JJ, López-Pousa A (2014). A randomized phase III trial comparing induction chemotherapy followed by chemoradiotherapy versus chemoradiotherapy alone in locally advanced head and neck cancer. Ann Oncol.

[16] Cooper JS, Zhang Q, Pajak TF (2012). Long-term follow-up of the RTOG 9501/intergroup phase III trial: postoperative concurrent radiation therapy and chemotherapy in high-risk squamous cell carcinoma of the head and neck. Int J Radiat Oncol Biol Phys.

[17] Posner MR, Hershock DM, Blajman CR (2007). Cisplatin and fluorouracil alone or with docetaxel in head and neck cancer. N Engl J Med.

[18] Lo Nigro C, Denaro N, Merlotti A (2017). Head and neck cancer: improving outcomes with a multidisciplinary approach. Cancer Manag Res.

